# Weighted Hypoxemia Index: An adaptable method for quantifying hypoxemia severity

**DOI:** 10.1371/journal.pone.0328214

**Published:** 2025-07-10

**Authors:** Diane C. Lim, Cheng-Bang Chen, Ankita Paul, Yujie Wang, Jinyoung Kim, Soonhyun Yook, Emily Y. Kim, Edison Q. Kim, Anup Das, Medhi Wangpaichitr, Virend K. Somers, Chi Hang Lee, Phyllis C. Zee, Toshihiro Imamura, Hosung Kim

**Affiliations:** 1 Department of Medicine, University of Miami, Miami, Florida, United States of America; 2 Department of Medicine, Miami VAHS, Miami, Florida, United States of America; 3 Department of Industrial and Systems Engineering, University of Miami, Coral Gables, Florida, United States of America; 4 Bioinformatics & Computational Biology, MD Anderson, Houston, Texas, United States of America; 5 School of Nursing, University of Nevada, Las Vegas, Nevada, United States of America; 6 Department of Neurology, Stevens Neuroimaging and Informatics Institute, University of Southern California, Keck School of Medicine, Los Angeles, California, United States of America; 7 Department of Research, Miami VAHS, Miami, Florida, United States of America; 8 Department of ECE, Drexel University, Philadelphia, Pennsylvania, United States of America; 9 Department of Cardiovascular Medicine, Mayo Clinic, Rochester, Minnesota, United States of America; 10 Department of Medicine, Yong Loo Lin School of Medicine, National University of Singapore, Singapore,; 11 Department of Cardiology, National University Heart Centre, Singapore, Singapore,; 12 Department of Neurology, Northwestern University, Chicago, Illinois, United States of America; 13 Department of Pediatrics, University of Pennsylvania, Philadelphia, Pennsylvania, United States of America; 14 Division of Pulmonary and Sleep Medicine, Children’s Hospital of Philadelphia, Philadelphia, Pennsylvania, United States of America; Charité - Universitätsmedizin Berlin, GERMANY

## Abstract

**Objective:**

To quantitate hypoxemia severity.

**Methods:**

We developed the Weighted Hypoxemia Index to be adapted to different clinical settings by applying 5 steps to the oxygen saturation curve: (1) Identify desaturation/resaturation event i by setting the upper threshold; (2) Exclude events as artifact by setting a lower threshold; (3) Calculate weighted area for each i, as ${(\Delta }_{i}\;\times \;\Phi_{i})$; (4) Calculate a normalization factor Ω for each subject; (5) Calculate the Weighted Hypoxemia Index as the summation of all weighted areas multiplied by Ω. We assessed the Weighted Hypoxemia Index predictive value for all-cause mortality and cardiovascular mortality using the Sleep Heart Health Study (enrollment 1995–1998, 11.1 years mean follow-up).

**Results:**

We set varying upper thresholds at 92%, 90%, 88%, and 86%, a lower threshold of 50%, calculated area under the curve and area above the curve, with and without a linear weighted factor (duration of each event i), and used the same normalization factor of total sleep time <90% divided by total sleep time. After excluding subjects with missing data, we analyzed 4,509 participants (Alive: N = 3,769; All-cause mortality: N = 1,071; cardiovascular mortality: N = 330). Since the Weighted Hypoxemia Index-Area Under the Curve set at upper threshold of 90% (WHI-AUC90) had the best results in predicting all-cause mortality, we then compared it to the Apnea-Hypopnea Index and Total Sleep Time <90%. WHI-AUC90 showed statistical significance across quintiles for all-cause mortality, but not cardiovascular mortality, in adjusted Cox regression models.

**Conclusion:**

The Weighted Hypoxemia Index offers a versatile and clinically relevant method for quantifying hypoxemia severity, with potential applications to evaluate mechanisms and outcomes across various patient populations.

## Introduction

Nocturnal hypoxemia, characterized by low S_p_O_2_ levels during sleep, is frequently evaluated in chronic disorders such as chronic obstructive pulmonary disease (COPD) [1], pulmonary hypertension [2], heart failure [3], and obstructive sleep apnea (OSA) [4–11]. Each condition has its own threshold for defining significant hypoxemia, making it challenging to evaluate the interaction between the lungs and heart in the setting of upper airway obstruction, which exacerbates hypoxemia.

OSA, diagnosed using the apnea-hypopnea index (AHI), is globally prevalent [12]. However, in December 2022, the Agency for Healthcare Research and Quality [13] concluded that not only are definitions of breathing measures inconsistent during sleep studies to diagnose OSA, but there is a lack of validity that the AHI can assess long-term health outcomes. While apneas and hypopneas may be the cause, frequently it is the severity of hypoxemia that correlates to outcomes. Measures of hypoxemia, such as the total sleep time under S_p_O_2_ of 90% (TST90) or minimum saturation (Min Sat) have been associated with increased cancer incidence [14,15] and mortality [16,17], atrial fibrillation [18], sudden cardiac death [19], and major adverse cardiac event (MACE) [20]. Unfortunately, TST90 quantifies time spent below 90%, but it does not reflect the severity of hypoxemia; Min Sat quantifies the severity of hypoxemia, but it does not reflect the temporal dimension of hypoxemia.

Several novel metrics have been developed to capture both duration and severity. Kulkas et al [21] developed several metrics including Desaturation Severity (DS), which is the summation of *area above* the S_p_O_2_ curve (AAC) for each event i then divided by total sleep time (TST); i is defined as an apnea/hypopnea event. This group demonstrated DS was significantly more associated with increased risk of daytime sleepiness [22] and increased risk of impaired psychomotor vigilance task performance [23] compared to AHI. Watanabe et al [24] developed the 4% Percent of Oxygen Desaturation events (4%POD) which is the summation of desaturation/resaturation *duration* for each event i then divided by total recording time (TRT); i is defined as a 4% oxygen desaturation event. They demonstrated 4%POD was an independent predictor of mortality in heart failure patients with central sleep apnea. They also calculated the summation of *area above* the S_p_O_2_ curve (AAC) divided by TRT. Lastly, they calculated Area Under the Threshold 90% (AUT90%), which is the summation of *area above* the S_p_O_2_ curve (AAC), but below 90% then divided by TRT. Both AAC and AUT90% did not predict mortality. Azarbarzin et al. [25] developed the Sleep Apnea Specific Hypoxic Burden (SASHB), where a search window was calculated using the average duration of all events i within a subject; i is defined as an apnea/hypopnea. Then for each event i, the *area above* the S_p_O_2_ curve (AAC) within the search window was calculated. All AAC were summed, then divided by TST. They demonstrated the higher quintiles can predict cardiovascular mortality [25] and incidence of heart failure in men [26]. Thanaviratananich et al. [27] developed the Hypoxemic Load under 100% (HL100) which is the entire *area above* the S_p_O_2_ curve (AAC) using 100% as a fixed upper threshold. They demonstrated HL100 was an independent predictor of fasting blood glucose.

Assuming hypoxemia is defined as an S_p_O_2_ below 90%, most novel metrics quantify both normoxemia and hypoxemia, except AUT90%. Hypoxemia, the delivery of less oxygen to the body, impacts intracellular metabolism and energy production, in that there is a shift from oxidative phosphorylation to glycolysis [28], depending on the organ (oxygen is preferentially delivered to the brain and heart) and duration of hypoxemia. When oxygen is not available, glycolysis produces less ATP [29] affecting cellular processes such as active transport [30], protein synthesis [31] and DNA repair [32]. Even when glycolysis preferentially occurs in the cytoplasm, the mitochondrial electron transport chain (ETS) is still active, but under hypoxic conditions, the reduction in available oxygen as the terminal electron acceptor causes electron backup in the ETC [33], leading to leakage and the formation of reactive oxygen species (ROS) [34]. In the setting of cyclical intermittent hypoxia, it is plausible that reoxygenation floods hypoxic cells with oxygen, which reacts with the leaked electrons and increase ROS production. Furthermore, this repeated, sudden influx of reoxygenation/ROS can overwhelm the cell’s antioxidant defenses [35] resulting in oxidative stress. Therefore, we present the Weighted Hypoxemia Index (WHI), a metric that allows investigators to use a weighted factor that “penalizes” severe drops in S_p_O_2_, the flexibility to calculate AAC or *area under* the S_p_O_2_ curve (AUC), set any upper threshold of hypoxemia, set the lower threshold to exclude artifact, and use a normalization factor to compare one subject to another.

## Methods

### Calculation of Weighted Hypoxemia Index

**Fig 1:**
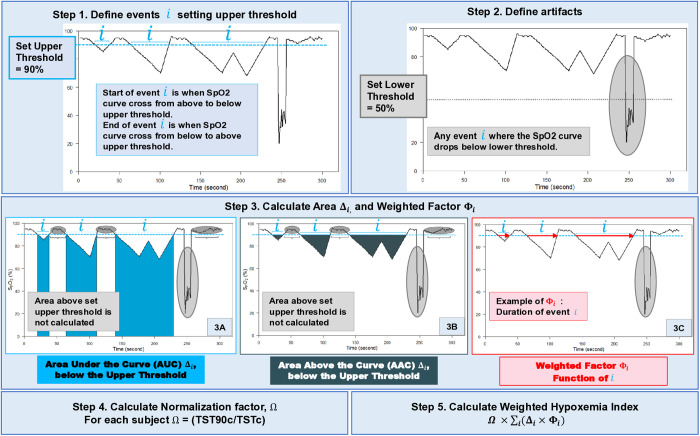
Weighted Hypoxemia Index (WHI). We developed five steps to calculate the WHI for each subject. Step 1 Define a desaturation/resaturation event, denoted as i, by setting the upper threshold. Step 2 Exclude artifacts by setting the lower threshold. Step 3 For each event i calculate area Δ, either (Panel 3A) Under the Curve (AUC) (light blue area) or (Panel 3B) Above the Curve (AAC) (dark blue area) and a weighted factor denoted as $\Phi_{i}$ (red arrow) (Panel 3C). Step 4 For each subject, calculate a normalization factor, denoted as Ω. TST90c is the total sleep time under 90%, corrected; TSTc is the total sleep time, corrected. Step 5 WHI is the sum of [$\left(\Delta_{i}\times \Phi_{i}\right)$ for each event i] then multiplied by Ω.

The WHI is calculated in five steps (Fig 1) using standardized 1 Hertz SpO2 signals as “the curve” and illustrated using data from the Sleep Heart Health Study (SHHS) (Table 1).

**Table 1:** Baseline characteristics by alive, all-cause mortality, and CVD mortality.

Total N = 4509	Alive N = 3438	ALL-Cause Mortality N = 1071	CVD Mortality N = 330
Age, mean (SD), years	61.6 (10.0)	73.8 (8.5)	75.7 (7.5)
Gender			
Female n(%)	1904 (55.38)	500 (46.69)	148 (44.85)
Male n(%)	1534 (44.62)	571 (53.31)	182 (55.15)
Race n(%)			
White	3021 (87.87)	950 (88.70)	285 (86.36)
Black	183 (5.32)	107 (9.99)	43 (13.03)
Other	234 (6.81)	14 (1.31)	2 (0.61)
BMI, mean (SD), kg/m2	28.4 (5.1)	27.8 (5.0)	27.5 (4.8)
COPD, n(%)	29 (0.84)	24 (2.24)	2 (0.61)
Diabetes, n(%)	171 (4.97)	162 (15.13)	70 (21.21)
Hypertension, n(%)	1189 (34.58)	647 (60.41)	233 (70.61)
Stroke, n(%)	83 (2.41)	82 (7.66)	35 (10.61)
MI, n(%)	148 (4.30)	160 (14.94)	74 (22.42)
Cardiac Revascularization, n(%)	141 (4.10)	140 (13.07)	59 (17.88)
Lipid lowering meds, n(%)	426 (12.39)	156 (14.36)	57 (17.27)
Smoking n(%)			
Never	1649 (47.96)	443 (41.36)	148 (44.85)
Former	318 (9.25)	98 (9.15)	22 (6.67)
Current	1471 (42.79)	530 (49.49)	160 (48.48)
Alcohol, n(%)			
< 1 drink/ week	1893 (55.06)	755 (70.49)	227 (68.79)
1–12 drinks/ week	1357 (39.47)	229 (21.39)	73 (22.12)
≥ 13 drinks/ week	188 (5.47)	87 (8.12)	30 (9.09)
Total sleep time, n(%)			
≤ 5 hours	284 (8.26)	102 (9.52)	41 (12.42)
5–8 hours	2911 (84.67)	830 (77.50)	241 (73.03)
≥ 8 hours	243 (7.07)	139 (12.98)	48 (14.55)
AHI, mean (SD), events/h	17.25 (15.34)	20.94 (17.44)	21.22 (16.55)
TST90, mean (SD), %	2.70 (8.32)	6.48 (15.35)	6.03 (15.12)
WHI–AUC90, n(%), %*sec^**2**^			
**Q1** (0–0.96)	789 (22.95)	113 (10.55)	40 (12.12)
**Q2** (0.96–18.31)	729 (21.20)	173 (16.15)	49 (14.85)
**Q3** (18.31–169.52)	685 (19.92)	216 (20.17)	72 (21.82)
**Q4** (169.52–2446.03)	660 (19.20)	242 (22.60)	79 (23.94)
**Q5** (2446.03−2,210,240)	575 (16.72)	327 (30.53)	90 (27.27)

#### Step 1. Define events i.

 Determine the upper threshold; an event i starts when the SpO2 curve crosses from above to below the upper threshold and ends when it crosses back to above the upper threshold. In Fig 1, as an example, we set the upper threshold at 90% with results in Table 2; comparisons of results when the upper threshold is set at 88%, 86%, and 92% are also provided in Table 2.

**Table 2:** WHI-AUC90 predicts All-Cause Mortality better than AHI and TST90.

	WHI-AUC90	Hazard Ratio	*P value*	AHI	Hazard Ratio	*P value*	TST90	Hazard Ratio	*P value*
**Model 0** *Metric Alone*	**Q1**	1	--	**Q1**	1	*--*	**Q1**	1	*--*
	**Q2**	1.63 (1.29, 2.07)	***<.001*** ^***^	**Q2**	1.12 (0.91, 1.39)	*.292*	**Q2**	0.94 (0.75, 1.18)	*.594*
	**Q3**	2.02 (1.61, 2.54)	***<.001*** ^***^	**Q3**	1.33 (1.08, 1.63)	***.007*** ^**^	**Q3**	1.37 (1.11, 1.68)	***.003*** ^**^
	**Q4**	2.46 (1.97, 3.07)	***<.001*** ^***^	**Q4**	1.65 (1.35, 2.01)	***<.001*** ^***^	**Q4**	1.48 (1.21, 1.82)	***<.001*** ^***^
	**Q5**	3.46 (2.79, 4.29)	***<.001*** ^***^	**Q5**	1.86 (1.53, 2.26)	***<.001*** ^***^	**Q5**	2.36 (1.95, 2.85)	***<.001*** ^***^
**Model 1** *Model 0 + Demographic*^a^ *+* *Cardiometabolic*^b^	**Q1**	1	*--*	**Q1**	1	*--*	**Q1**	1	*--*
	**Q2**	1.27 (1.00, 1.62)	***.047*** ^*^	**Q2**	0.82 (0.66, 1.02)	*.078*	**Q2**	1.00 (0.80, 1.25)	*.973*
	**Q3**	1.28 (1.02, 1.62)	***.036*** ^*^	**Q3**	0.89 (0.72, 1.10)	*.289*	**Q3**	1.11 (0.90, 1.37)	*.332*
	**Q4**	1.37 (1.09, 1.72)	***.007*** ^**^	**Q4**	0.86 (0.70, 1.06)	*.166*	**Q4**	1.11 (0.90, 1.37)	*.308*
	**Q5**	1.69 (1.35, 2.11)	***<.001*** ^***^	**Q5**	0.97 (0.78, 1.19)	*.756*	**Q5**	1.46 (1.20, 1.79)	***<.001*** ^***^

**Model 0** is unadjusted hazard ratios (HR) for Weighted Hypoxemia Index (WHI).

**Model 1** is adjusted for Demographic and Cardiometabolic covariates.

^a^Demographic covariates include age, gender, race, BMI, COPD, smoking, alcohol, and sleep duration.

^b^Cardiometabolic covariates include diabetes, hypertension, congestive heart failure, angina, myocardial infarction, coronary revascularization, stroke, lipid-lowering medication.

**WHI-AUC90,** Weighted Hypoxemia Index of Area Under the Curve set at upper threshold of 90%.

**AHI,** Apnea-hypopnea index, 3% criterion.

**TST90,** percent time of study with oxygen saturation below 90%.

**Min Sat**, minimum saturation.

* *P* < .05; ^**^ *P* < .01; ^***^ *P* < .001. Quintiles 2–5 are compared to Quintile 1.

#### Step 2. Define artifacts.

Determine the lower threshold. Any event i where the S_p_O_2_ curve drops below the lower threshold will be excluded. In Fig 1, as an example, we set the lower threshold at 50%, since most pulse oximeters are calibrated between 100% and 70% [36].

#### Step 3. Calculate area and weighted factor for each event i.

*Area (*$\Delta_{i}$*)* Calculate the AUC or AAC using the trapezoidal rule. For events where the area is both below (panel 3A) and above (panel 3B) the upper threshold, only areas below the upper threshold are included; any event entirely above the upper threshold is not included. Fig 1 demonstrates both AUC (3A) and AAC (3B), both with the upper threshold set at 90%. *Weighted Factor,* $\Phi_{i}$ While a nonlinear weighted factor may better explain heterogeneity in biological outcome, the determination of a nonlinear factor would require validation with a large dataset to prevent overfitting and may lead to poor generalization. Therefore, since it has been demonstrated that event duration is significant in the setting of OSA [37,38], we used a linear weighted factor that is the duration of i (panel 3C). Results with a weighted factor compared to results without a weighted factor is provided in Table 2.

#### Step 4. Calculate normalization factor Ω.

To allow between-subject comparisons, a normalization factor is needed. For our example, we calculated the **c**orrected total sleep time below 90%, excluding artifacts (TST90**c**) divided by the **c**orrected total sleep time (TST**c**). Start time of sleep is defined as three consecutive N1/N2/N3, and end of sleep is defined as the last N1/N2/N3/REM. We used the manual sleep stage annotations from SHHS to be consistent with their start/end to determine AHI and TST90 quintiles.

#### Step 5. Calculate WHI.

For each event i, multiply the area ($\Delta_{i};\;$units: seconds×%) by its weighted factor ($\Phi_{i};\;$unit: seconds); then sum all events i; then multiply by the normalization factor (Ω; unitless). This is the WHI index (sec^2^×%).

### Description of Sleep Heart Health Study (SHHS)

**Fig 2:**
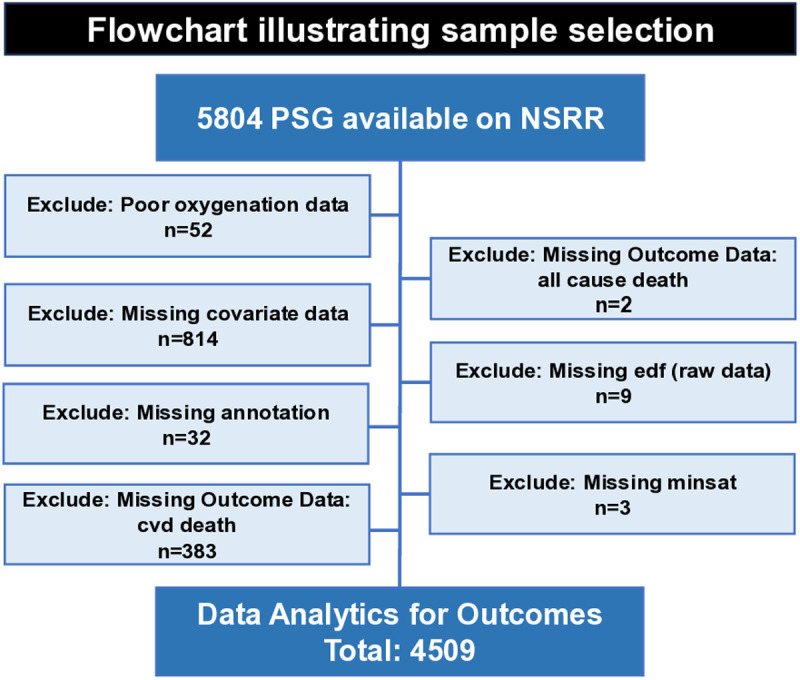
Flowchart illustrating sample selection from Sleep Heart Health Study (SHHS). To illustrate the utility of the WHI in a clinical setting, we leveraged the SHHS, a database that is publicly available. This flowchart broadly outlines exclusion of subjects; specific variable names used to exclude subjects are listed in S1 Fig. Our primary outcome was all-cause mortality, and our secondary outcome was CVD mortality. PSG = polysomnogram; NSRR = National Sleep Research Resource; CVD = cardiovascular disease; edf = European Data Format; minsat = Minimum Oxygen Saturation.

This study involves secondary analysis of a publicly available, de-identified dataset, available on the National Sleep Research Resource website, https://sleepdata.org/, accessed on August 1, 2022; authors did not have access to information that could identify subjects during or after data collection. No new data collection involving human participants was conducted. To demonstrate whether the WHI can predict all-cause mortality and cardiovascular mortality, we used longitudinal data from the SHHS [39,40], a multi-center observational community cohort sponsored by the National Heart Lung and Blood Institute to determine cardiovascular and other consequences of sleep-disordered breathing. Methodological details of the study have been reported previously [25,40,41]. In short, 6,441 men and women aged ≥40 years old were enrolled between 1995 and 1998 that included a baseline PSG. Outcome data was collected between baseline and 2011, for an average of 11.1 years of follow-up. ***Subject selection*** Among 6,441 baseline participants, 5804 were publicly available on the National Sleep Research Resource (NSRR) website. Fig 2 is a flowchart illustrating how we excluded subjects. Additional details regarding specific variable names that were used to determine exclusion can be found in S1 Fig in the online Supplement. Thus, a final sample of 4,509 subjects, N = 3,769 Alive, N = 1,071 All-cause mortality and a subset of all-cause mortality, cardiovascular disease (CVD) mortality, N = 330, was used for data analytics (Table 1).

### Statistical analyses

Statistical analyses were conducted using R Version 4.3.1, while data processing was performed using Python 3.9.18. To compare the WHI to the AHI and TST90, values were ranked in ascending order then categorized into quintiles with each quintile representing 20% of the data. The lowest quintile (Quintile 1, 0–20%) served as the reference group.

To validate the predictive value of WHI, we compared it with AHI and TST90. To test whether the WHI, AHI or TST90 can predict all-cause mortality or CVD mortality, we employed several models. First, Model 0 evaluated each metric independently (unadjusted) and Model 1 was adjusted for clinically relevant demographic and cardiometabolic covariates (Table 3). Demographic covariates consisted of age, gender, race, body mass index (BMI), COPD, smoking status (never, former, current), alcohol consumption (<1, 1–12, > 12) and sleep duration (≤5h, 5-8h, ≥ 8h). Cardiometabolic covariates consist of baseline history of diabetes, hypertension, congestive heart failure, angina, myocardial infarction, coronary revascularization, stroke, and use of lipid-lowering medication. Binary, categorical, and continuous variables were appropriately managed to mitigate multilinearity concerns, ensuring variance inflation factors remained below five [42]. The Cox regression models were used to determine the Hazard Ratio for all-cause mortality based on each metric and covariates. Second, additional hierarchical models (1A, 2A, 2B, 3 and 4) are presented in S2 and S3 Tables, detailed in the online Supplement. To account for multiple testing across biomarkers and quintiles, we applied Benjamini-Hochberg correction to the p-values for Tables 3 and S1–S3 (adjusted p-values available in Supporting Information S4–S7 Tables).

**Table 3:** Model 1 predicting All-Cause mortality, comparison of (1) weighted vs unweighted, and (2) AUC vs AAC (3) at different set upper thresholds.

Weighted	Quintiles	Hazard Ratio	*P value*	Unweighted	Quintiles	Hazard Ratio	*P value*
**WHI** **AUC** **92**	**Q1**	1	*--*	**AUC** **92**	**Q1**	1	*--*
	**Q2**	1.22 (0.96, 1.56)	*.108*		**Q2**	1.35 (1.05, 1.72)	***.018*** ^*^
	**Q3**	1.19 (0.94, 1.52)	*.152*		**Q3**	1.25 (0.98, 1.60)	*.076*
	**Q4**	1.13 (0.88, 1.45)	*.333*		**Q4**	1.24 (0.97, 1.59)	*.091*
	**Q5**	1.57 (1.24, 2.00)	***<.001*** ^***^		**Q5**	1.58 (1.24, 2.01)	***<.001*** ^***^
**WHI** **AAC** **92**	**Q1**	1	*--*	**AAC** **92**	**Q1**	1	*--*
	**Q2**	1.36 (1.06, 1.74)	.017^*^		**Q2**	1.32 (1.03, 1.69)	***.027*** ^*^
	**Q3**	1.33 (1.04, 1.70)	.022^*^		**Q3**	1.29 (1.01, 1.65)	***.039*** ^*^
	**Q4**	1.25 (0.97, 1.60)	*.081*		**Q4**	1.29 (1.01, 1.66)	***.040*** ^*^
	**Q5**	1.68 (1.32, 2.14)	*<.* ***001*** ^***^		**Q5**	1.58 (1.24, 2.01)	***<.001*** ^***^
**WHI** **AUC** **90**	**Q1**	1	*--*	**AUC** **90**	**Q1**	1	*--*
	**Q2**	1.27 (1.00, 1.62)	***.047*** ^*^		**Q2**	1.26 (0.99, 1.59)	*.060*
	**Q3**	1.28 (1.02, 1.62)	***.036*** ^*^		**Q3**	1.29 (1.02, 1.62)	***.033*** ^*^
	**Q4**	1.37 (1.09, 1.72)	***.006*** ^**^		**Q4**	1.34 (1.07, 1.68)	***.011*** ^*^
	**Q5**	1.69 (1.35, 2.11)	***<.001*** ^***^		**Q5**	1.66 (1.32, 2.07)	***<.001*** ^***^
**WHI** **AAC** **90**	**Q1**	1	*--*	**AAC** **90**	**Q1**	1	*--*
	**Q2**	1.27 (1.00, 1.61)	***.053***		**Q2**	1.25 (0.99, 1.59)	*.064*
	**Q3**	1.38 (1.10, 1.74)	***.006*** ^**^		**Q3**	1.32 (1.05, 1.67)	***.018*** ^*^
	**Q4**	1.39 (1.11, 1.74)	***.005*** ^**^		**Q4**	1.37 (1.09, 1.72)	***.006*** ^**^
	**Q5**	1.73 (1.38, 2.17)	***<.001*** ^***^		**Q5**	1.71 (1.37, 2.14)	***<.001*** ^***^
**WHI** **AUC** **88**	**Q1**	1	*--*	**AUC** **88**	**Q1**	1	*--*
	**Q2**	1.31 (1.03, 1.66)	***.027*** ^*^		**Q2**	1.37 (1.08, 1.73)	***.009*** ^**^
	**Q3**	1.48 (1.18, 1.85)	***<.001*** ^***^		**Q3**	1.43 (1.14, 1.79)	***.002*** ^**^
	**Q4**	1.57 (1.26, 1.96)	***<.001*** ^***^		**Q4**	1.54 (1.24, 1.92)	***<.001*** ^***^
	**Q5**	1.72 (1.39, 2.14)	***<.001*** ^***^		**Q5**	1.76 (1.41, 2.18)	***<.001*** ^***^
**WHI** **AAC** **88**	**Q1**	1	*--*	**AAC** **88**	**Q1**	1	*--*
	**Q2**	1.31 (1.04, 1.66)	***.024*** ^*^		**Q2**	1.34 (1.05, 1.69)	***.017*** ^*^
	**Q3**	1.46 (1.17, 1.82)	***<.001*** ^***^		**Q3**	1.47 (1.17, 1.83)	***<.001*** ^***^
	**Q4**	1.57 (1.26, 1.96)	***<.001*** ^***^		**Q4**	1.56 (1.25, 1.94)	***<.001*** ^***^
	**Q5**	1.74 (1.40, 2.16)	***<.001*** ^***^		**Q5**	1.73 (1.39, 2.15)	***<.001*** ^***^
**WHI** **AUC** **86**	**Q1**	1	*--*	**AUC** **86**	**Q1**	1	*--*
	**Q2**	0.83 (0.63, 1.09)	*.181*		**Q2**	0.83 (0.63, 1.09)	*.181*
	**Q3**	1.21 (1.00, 1.46)	***.047*** ^*^		**Q3**	1.22 (1.01, 1.46)	***.041*** ^*^
	**Q4**	1.30 (1.08, 1.56)	***.005*** ^**^		**Q4**	1.29 (1.07, 1.54)	***.007*** ^**^
	**Q5**	1.40 (1.17, 1.68)	***<.001*** ^***^		**Q5**	1.41 (1.18, 1.69)	***<.001*** ^***^
**WHI** **AAC** **86**	**Q1**	1	*--*	**AAC** **86**	**Q1**	1	*--*
	**Q2**	0.83 (0.63, 1.09)	*.181*		**Q2**	0.83 (0.63, 1.09)	*.183*
	**Q3**	1.18 (0.98, 1.42)	*.083*		**Q3**	1.23 (1.02, 1.48)	***.028*** ^*^
	**Q4**	1.32 (1.10, 1.58)	***.003*** ^**^		**Q4**	1.28 (1.07, 1.54)	***.007*** ^**^
	**Q5**	1.41 (1.18, 1.69)	***<.001*** ^***^		**Q5**	1.40 (1.17, 1.67)	***<.001*** ^***^

**WHI** are metrics that are weighted using a linear weighted factor of duration of desaturation/resaturation event.

**AUC** are metrics where area is calculated under the S_p_O_2_ curve.

**AAC** are metrics where area is calculated above the S_p_O_2_ curve.

**The number** following AUC/AAC is the upper threshold, e.g., AAC88 is the area above the curve below an upper threshold of 88%.

**Model 1** uses the same 4509 subjects characterized in Table 1: Hazard ratios (95% confidence intervals) controlled for demographic covariates (age, gender, race, BMI, COPD, smoking, alcohol, sleep duration) and cardiometabolic covariates (diabetes, hypertension, congestive heart failure, angina, myocardial infarction, coronary revascularization, stroke, lipid-lowering medication).

* *P* < .05; ^**^ *P* < .01; ^***^ *P* < .001. Quintiles 2–5 are compared to Quintile 1.

**Fig 3:**
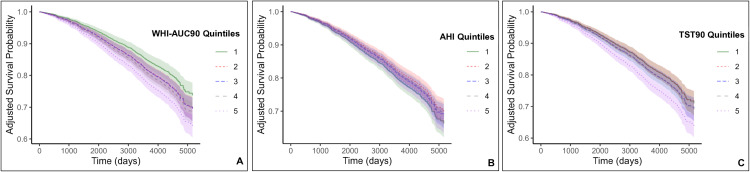
Adjusted predicted survival curve for Model 1 depicting all-cause mortality. The survival probability is presented for Model 1 (Hazard Ratios and confidence intervals in Table 2), comparing (A) WHI-AUC90 (Weighted Hypoxemia Index-Area Under Curve, threshold set at 90%), (B) AHI (Apnea-Hypopnea Index) and (C) TST90 (Total Sleep Time, Oxygen Saturation under 90%) quintiles. Model 1 encompasses Model 0, demographic covariates (age, gender, race, BMI, COPD, smoking, alcohol, sleep duration) and cardiometabolic covariates (diabetes, hypertension, congestive heart failure, angina, myocardial infarction, coronary revascularization, stroke, lipid-lowering medication). Adjusted survival curves were computed by averaging the predicted survival curves for each subject within the SHHS cohort.

Survival curves for WHI, AHI and TST90 quintiles for all-cause mortality were generated utilizing Model 1 (Fig 3). Individual survival curves were obtained for all 4,509 subjects using a Cox regression for Hazard Ratio and were subsequently averaged for composite survival curve depiction [43]. Additional survival curves for all-cause mortality, Model 4 are presented in S2 Fig in the online Supplement.

## Results

### Baseline characteristics of SHHS

Clinical characteristics of SHHS subjects are summarized in Table 1. Among 4,509 evaluated subjects, N = 1,071 were assessed for all-cause mortality (mean age 73.8 years ± 8.5) and N = 3,438 subjects were assessed for alive (mean age 61.6 years ± 10.0). A subset of all-cause mortality, CVD mortality, was also assessed (mean age 75.7 years ± 7.5).

Since the weighted hypoxemia index using AUC with an upper SpO2 threshold of 90% (WHI-AUC90) demonstrated the strongest association with mortality in preliminary analyses, we selected this measure for detailed comparison against traditional metrics. We subsequently conducted a comprehensive analysis comparing WHI-AUC90 to the AHI and TST90, using quintile groupings for each metric, to evaluate their respective abilities to predict all-cause mortality and cardiovascular mortality. The WHI-AUC90 ranged from 0 to 2,210,240, which was ranked in order, then divided into quintiles (Table 1): Quintile 1 is 0–0.96, Quintile 2 is 0.96–18.31, Quintile 3 is 18.31–169.52, Quintile 4 is 169.52–2,446.03, Quintile 5 is 2,446.03–2,210,240. The AHI, taken directly from the SHHS database using the 3% rule, ranged from 0 to 161.84 events/hour, which was ranked then quintiles (Table 1): Quintile 1 is 0–5.77, Quintile 2 is 5.77–10.57, Quintile 3 is 10.57–17.00, Quintile 4 is 17.00–27.64, Quintile 5 is 27.64–161.84. The Total Sleep Time under oxygen saturation of 90% (TST90), taken directly from the SHHS database, ranged from 0 to 100%, which was ranked then quintiles (Table 1): Quintile 1 is 0–0, Quintile 2 is 0–0.08, Quintile 3 is 0.08–0.52, Quintile 4 is 0.52–2.92, Quintile 5 is 2.92–100.

### WHI-AUC90 predicts all-cause mortality

We employed both unadjusted (Model 0) and adjusted (Model 1) analysis, quantified through quintile-specific likelihood (hazards ratio, HR) (Table 2). For all-cause mortality, while all three metrics displayed significance when unadjusted (Model 0), it is the WHI-AUC90 that demonstrates significance across all quintiles when adjusted for demographic and cardiometabolic covariates (Model 1). Specifically, Q2 (HR = 1.27, CI = 1.00–1.62, *p = .047*); Q3 (HR = 1.28, CI = 1.02–1.62, *p = .036*); Q4 (HR = 1.37, CI = 1.09–1.72, *p = .007*); Q5 (1.69, CI = 1.35–2.11, *p < .001*). We plotted the survival curve (Fig 3) of WHI-AUC90 using Model 1. Adjusted survival curves were computed by averaging the predicted survival curves for each subject within the SHHS cohort, then averaged by quintile. To demonstrate how all three metrics (WHI-AUC90, AHI, TST90) performed in alternative models, we conducted five additional models presented in S1 Table. Additional survival curves for Model 4 are presented in S2 Fig.

Next, utilizing raw signals from the pulse oximeter of the PSG, we calculated hypoxemia using AUC and AAC with a set upper threshold of 92%, 90%, 88% and 86%, with and without a weighted factor. We then calculated the weighted and unweighted hypoxemia index for 4,509 subjects. We present comparative analysis of the adjusted Model 1 to predict all-cause mortality through quintile-specific likelihood (hazards ratio, HR) in Table 3.

### WHI does not predict cardiovascular mortality

We present Models 0–4 of **WHI-AUC90** in S2 Table; none were able to predict CVD mortality. We present comparative analysis of the adjusted Model 1 to predict cardiovascular mortality using AUC and AAC at upper thresholds of 92%, 90%, 88% and 86%, both weighted and unweighted in S3 Table.

## Discussion

The strength of the WHI, the metric, lies in its adaptability; investigators can set upper and lower thresholds, calculate AUC or AAC, use the weighted factor or not, and determine an appropriate normalization factor according to their study. Using a model of OSA to predict all-cause mortality, after adjustment for demographic and cardiometabolic factors (Table 2, Model 1), the AHI and TST90 were no longer significant predictors of mortality within the SHHS cohort, whereas the WHI remained significant. This is likely because the mortality risk in Model 0 using AHI or TST90 may be explained by underlying factors (e.g., older age, higher BMI) rather than the metrics themselves. In contrast, the WHI, which integrates the depth and duration of hypoxemia below the upper threshold, is an independent predictor of all-cause mortality even after full adjustment. This suggests that the WHI quantitates hypoxemia severity that more directly links physiological hypoxemia to mortality risk, beyond conventional OSA metrics (S1 Table). In addition, the results were most significant with upper thresholds set at 90% or 88%, less significant at 86%, and even less so at 92%. This is further supported by Hazard Ratios, which increased sequentially from Q2 to Q5 at 90%, 88%, and 86% but not at 92%. The variation in results between weighted and unweighted settings likely stems from the linear weighted factor used, with higher thresholds potentially overestimating and lower thresholds underestimating hypoxemia. Notably, the Hazard Ratio was most significant with a weighted factor and an upper threshold set at 90%.

Interestingly, the WHI did not significantly predict cardiovascular-specific mortality in our cohort. One potential explanation is an “observer effect” where subjects with significant hypoxemia were worked up and treated for cardiovascular disease. Another potential explanation is that well-controlled comorbidities such as blood pressure control, or revascularization treatments may have a stronger impact on preventing CVD mortality compared to the hypoxemia measured once at baseline.

### Limitations

Hypoxemia may be a physiological biomarker that reflects underlying factors and mechanisms, similar to how a single morning glucose level reflects many underlying mechanisms from insulin sensitivity to muscle utilization of glucose. Furthermore, one WHI measurement is unlikely to predict specific clinical consequences, again, similar to how a single morning glucose level cannot determine the risk of future amputation or heart disease. However, repeated WHI assessments, when combined with other relevant data, could offer valuable insights. Our results from the SHHS cohort were not adjusted for positive airway pressure usage or medication adherence, which may account for the WHI’s lack of predictive power for cardiovascular mortality. In addition, the SHHS is an older dataset which does not reflect contemporary CVD treatment or risk factors. We chose this dataset because it is publicly available, well-curated, and contains long-term outcomes that enable us to develop a model to demonstrate the association of hypoxemia severity and all-cause mortality. It is possible that other factors, such as the effectiveness of blood pressure medications, play a more critical role in CVD mortality than a single baseline measure of hypoxemia. Since the WHI did predict all-cause mortality in the SHHS cohort, this may be due to hypoxemia in older subjects serving as an indicator of overall health or “reserve” encompassing underlying chronic conditions such as COPD or heart failure. To further validate the utility of the WHI, especially in overlap syndromes such as OSA with COPD or CHF, validation across different databases that include younger subjects, and potentially, revisions to the weighted factor, are necessary.

### Future direction

Measurement of oxygen saturation of peripheral blood (S_p_O_2_) is ubiquitously utilized, from neonates [44] to adults, from the intensive care unit (ICU) to wearables [45]. Currently there is no method of comparing cumulative hypoxemia across different clinical, preclinical and in vitro settings. In the era of precision medicine, it is likely that we will shift towards using different thresholds and different weighted factors to calculate the WHI, but it would be useful to do this in a way that is comparable. For example, adjusting thresholds and factors from childhood to adult could assess an individual’s “reserve” within oneself over time (e.g., AAC using 100% as the set upper threshold), but also comparability to other people (e.g., AUC) to provide insights into disease management. Furthermore, while the metric WHI reflects biology (e.g., one’s genetics + environmental factors), it is likely that different models will need to be developed depending on the syndrome/disease evaluated. For example, if evaluating obesity hypoventilation syndrome (OHS), different inputs such as effect of carbon dioxide on hypoxemia, would be added as covariates to the model. Therefore, we have identified a growing list of future studies:

Our first objective is to collaborate with the scientific community to mathematically capture the biological consequences of hypoxemia, spanning from in vitro studies to preclinical models, clinical trials, and population studies that integrate mechanistic data with clinical relevance. For instance, in the context of pulmonary hypertension, we might correlate WHI trends with specific outcomes, such as metabolic alterations, when comparing the efficacy of drug X versus drug Y for an individual. Since the principles underlying the WHI can be applied to monitoring devices like Near-Infrared Spectroscopy or End-Tidal CO2 monitoring, a combined metric in the laboratory could enhance the assessment of treatment. However, given that the WHI can be derived from wearable devices, WHI can also provide continuous monitoring of treatment effectiveness as patients go about their daily activities—awake versus asleep, exercising versus resting, or in combination with different clinical conditions. Moreover, WHI trends can help identify triggers, allowing for the prediction and prevention of exacerbations. This continuous feedback can also motivate patients to adhere to treatment, similar to how glucose readings provide feedback on insulin timing to meals, or the effects of not eating the donut and exercising instead.

Second, while we used a linear weighted factor (duration of each event), based on the hemoglobin desaturation curve, exploring a nonlinear weighted factor may improve the accuracy of our results. The optimal nonlinear factor would likely depend on the specific cellular outcome deemed significant. For example, some may prioritize intracellular hypoxia leading to increased lactic acid, while others may focus on hypoxia-induced HIF stabilization and downstream effects of a specific gene. As we refine clinically significant nonlinear weighted factors, linking clinical outcome data to the WHI could provide valuable insights into which molecular mechanisms are indicative of “overall reserve” versus specific outcomes.

Third, while the WHI, AHI and TST90 was unable to predict CVD mortality, other investigators correlated measures of hypoxemia to specific clinical outcomes, such as incidence of atrial fibrillation [18,46,47], heart failure [3,24,26], sudden cardiac death [19], and cancer [14]. In the future we will directly compare the WHI to other metrics of hypoxemia to predict the incidence of specific clinical outcomes, effect of circadian time and gender differences, and use of machine learning to predict mortality.

## Conclusion

This study presents and illustrates the weighted hypoxemia index, which can be adapted to different thresholds, AUC vs AAC, use of the weighted factor or not, and to different outcomes (all-cause mortality vs cardiovascular mortality). Collectively, results provide different insights that lead to interesting questions to step us closer to precision medicine.

## Supporting information

S1 File Supplement. This Supplement provides additional details of applying the weighted hypoxemia index (WHI), specifically: (1) Rationale: Excluding Subjects by Variable; (2) Pre-processing Raw Pulse Oximeter Signals; (3) Alternative Models For All-Cause Mortality; (4) WHI Does Not Predict CVD Mortality; (5) Adjusted p-values of Tables 3 and S1–S3.
(DOCX)

S1 Fig Detailed flowchart illustrating sample selection from Sleep Heart Health Study. To ensure reproducibility, we list the variables utilized for subjects at baseline assessment (Visit 1) that were excluded. Poor oxygenation signals were excluded using the variable [restan5]; subjects missing all-cause mortality data were excluded using the variable [vital]; subjects missing covariate data were excluded in this order: age [age_s1], gender [gender], race [race], BMI [bmi_s1], COPD [copd15], smoking status [smokestat_s1], sleep duration [hrswd02], hypertension [htnderv_s1], diabetes [parrptdiab], congestive heart failure [hf15], angina [angina15], myocardial infarction [mi15], coronary revascularization [ca15 + cabg15], stroke [stroke15], lipid-lowering medication [lipid1], alcohol use [alcohol]; subjects with missing edf or annotation files or missing minsat on the report were excluded; subjects with missing cardiovascular mortality data were excluded using the variable [cvd_death].
(TIF)

S2 Fig Adjusted predicted survival curve Model 4 depicting all-cause mortality. The survival probability for Model 4 (Hazard Ratios and confidence intervals in S1 Table) is presented for (A) WHI-AUC90, (B) AHI, and (C) TST90 models. Model 4 is adjusted for: (1) demographic covariates (age, gender, race, BMI, COPD, smoking, alcohol, sleep duration), (2) cardiometabolic covariates (diabetes, hypertension, congestive heart failure, angina, myocardial infarction, coronary revascularization, stroke, lipid-lowering medication), (3) AHI (for WHI-AUC90 and TST90 models), (4) TST90/minsat (for WHI-AUC90 and AHI models) and (5) WHI-AUC90 (for AHI and TST90 models). Adjusted survival curves were computed by averaging the predicted survival curves for each subject within the SHHS cohort. Note that among the survival curves by quintile the WHI-AUC90 demonstrates a coherent and logical progression specifically in comparison to AHI.
(TIF)

S1 Table Weighted Hypoxemia Index predicts All-Cause Mortality better than TST90 and AHI. (DOCX)

S2 Table Weighted Hypoxemia Index does not predict CVD mortality. (DOCX)

S3 Table Model 1 predicting CVD mortality, comparison of weighted vs unweighted and AUC vs AAC at different upper thresholds. (DOCX)

S4 Table Benjamini-Hochberg correction of Table 3. (DOCX)

S5 Table Benjamini-Hochberg correction of S1 Table. (DOCX)

S6 Table Benjamini-Hochberg correction of S2 Table. (DOCX)

S7 Table Benjamini-Hochberg correction of S3 Table. (DOCX)
