# Supplementary material for: Weighted Hypoxemia Index: An adaptable method for quantifying hypoxemia severity
Source: PLoS One. 2025 Jul 10;20(7):e0328214. doi: 10.1371/journal.pone.0328214 (PMC12244826; doi:10.1371/journal.pone.0328214)
Supplement: S1 File — This Supplement provides additional details of applying the weighted hypoxemia index (WHI), specifically: (1) Rationale: Excluding Subjects by Variable; (2) Pre-processing Raw Pulse Oximeter Signals; (3) Alternative Models For All-Cause Mortality; (4) WHI Does Not Predict CVD Mortality; (5) Adjusted p-values of Tables 3 and S1–S3. (DOCX) [file pone.0328214.s001.docx]

**Supplement**

This Supplement provides additional details of applying the weighted hypoxemia index (WHI), specifically:

1. Rationale: Excluding Subjects by Variable
2. Pre-processing Raw Pulse Oximeter Signals
3. Alternative Models For All-Cause Mortality
4. WHI Does Not Predict CVD Mortality
5. Adjusted p-values of Tables 3, S1, S2, S3
6. **Rationale: Excluding Subjects by Variable**

Excluding subjects can dramatically affect results, therefore, when excluding subjects, every effort was made to be unbiased. The National Sleep Research Resource is a remarkable database and efforts to harmonize data and variables are commendable. Once within the Sleep Heart Health Study (SHHS) database <https://sleepdata.org/datasets/shhs> there are 3 tabs, “Docs,” “Files,” and “Variables.” We found excluding subjects by variable name requires the expertise of both a clinician and a data scientist to understand the data dictionary and exclude the subject from the final dataset.

In **S1 Fig**, we report the order of how we excluded subjects using the specific variable name (provided in between [xxx]), domain (reported by the SHHS group as “1” or “NA” or “0” which is the threshold for how we determined which subjects to exclude), and how many subjects were excluded for each variable/domain (provided as “n=xx”). The rational for excluding these subjects is as follows: (1) Poor oxygenation data: Since the WHI metric is completely dependent on oxygenation data, rather than arbitrarily determine who had poor oxygenation data, we used the variable name “restan5” which was determined by the sleep technician that scored the study to have bad oximetry signals: “*Polysomnography (PSG) Scoring Notes (Sleep Heart Health Study Visit One (SHHS1)): Intervening period bad Resp./Oximetry*”; if the domain was “*1:Yes*” we excluded the subject, which was a total of 52 subjects. (2) Missing outcome data, Missing covariate data: Since the model we developed was to predict all-cause mortality, including CVD mortality, a subset of all-cause mortality, if the outcome data is missing, we excluded the subject. Since the model also considered covariates, if a subject had missing data, they were also excluded. (3) Missing edf, missing annotation files and missing minsat: Obviously, Missing edf: without any pulse ox data, WHI could not be calculated; Missing annotation files: this was necessary to determine start and end of a study; Missing minsat on the report: this was used to adjust the final models. A total of 4509 subjects were used to generate models for all-cause mortality and CVD mortality applied to all quintiles.

1. **Pre-processing Raw Pulse Oximeter Signals**

The importance of pre-processing raw pulse oximeter signals is to allow for comparison across different datasets and different technologies. Specifically, the following concepts should be considered: (1) Artifact removal: By using the lower threshold to exclude artifact, there is no need to additionally “clean” the data. (2) Area and Weighted factor: both are dependent on standardizing the hertz of the pulse oximeter signal. While a higher hertz may offer higher resolution of trapezoid calculation, we used 1Hz. (3) Normalization: both TST90c and TSTc required knowing when the study “starts” and “ends.” We tested several variations of start and there was almost no difference. In the end, we selected 3 consecutive N1 or N2 or N3 as the start to be consistent with other studies. After requesting access to the SHHS on the National Sleep Research Resource (NSRR) website <https://sleepdata.org/> we downloaded raw pulse oximeter signals from the baseline polysomnogram in European Data Format (edf). Since the SHHS pulse oximeter signals were sampled at 1Hz, there was no need to harmonize the data. If calculating the WHI using signals sampled at rates lower or higher than 1Hz, we recommend signals be standardized to 1Hz. As wearable technologies become more a part of our daily life and eventually, clinical studies, battery life becomes a tradeoff. Therefore, whether to normalize to 1Hz or a lower rate is a feature that can be adapted when calculating the WHI but communicating this will facilitate effective scientific communication and translational research between basic science and clinical practice.

1. **Alternative Models For All-Cause Mortality**

In Model 1, we adjusted for demographic and cardiometabolic covariates mirroring the analytical approach typically employed by physicians to assess data significance and its impact on outcomes. However, we conducted several more models to address other questions. In each subsequent model, we assessed the predictive capacity of the WHI-AUC90 for all-cause mortality using the same subjects grouped per quintile reported in Model 0 and Model 1. Notably, different combinations of covariates are presented to understand the impact of each factor on the outcome in **S1 Table**. Model 1A is adjusted for the metric (WHI-AUC90 or AHI or TST90) and demographic data (age, gender, race, BMI, COPD, smoking status, alcohol quantity and sleep duration). Here, we demonstrate HR is statistically significant for WHI-AUC90 across all quintiles, but only for quintile 5 for TST. When investigating the potential of the WHI-AUC90 to augment prediction beyond AHI (Model 2A), TST90+Min Sat (Model 2B), or AHI+TST90+Min Sat (Model 3), the WHI is able to predict all-cause mortality. This is evidenced by the statistically significant hazard ratio (HR) observed consistently across all quintiles. Lastly, when asking whether the WHI-AUC90 can provide additional data to predict all-cause mortality when controlling for both demographic and cardiometabolic (diabetes, hypertension, congestive heart failure, angina, myocardial infarction, coronary revascularization, stroke, lipid-lowering medication use) covariates as well as AHI+TST90+Min Sat (Model 4), again, WHI-AUC90 continues to demonstrate statistical significance across all quintiles. We plotted survival curves for Model 4 in **S2 Fig**.

1. **WHI-AUC90 Does Not Predict CVD Mortality**

When investigating the potential of the WHI-AUC90 to predict cardiovascular mortality, we generated Models 0-4 (**S2 Table**). Other than unadjusted Model 0, WHI, AHI and TST90 were not able to predict CVD mortality.

Utilizing raw signals from the pulse oximeter of the PSG, we calculated hypoxemia using AUC and AAC with a set upper threshold of 92%, 90%, 88% and 86%, both weighted and unweighted. We then calculated the WHI for 4,509 subjects and employed adjusted Model 1 analysis to evaluate their predictive potential for CVD mortality in **S3 Table**.

**5. Adjusted p-values of Tables 3, S1, S2, S3**

To account for multiple testing across biomarkers and quintiles, we applied Benjamini-Hochberg correction to the p-values for Tables 3 (**S4 Table**), S1 (**S5 Table**), S2 (**S6 Table**), S3 (**S7 Table**).
