# Supplementary figures and images for: Weighted Hypoxemia Index: An adaptable method for quantifying hypoxemia severity
Source: PLoS One. 2025 Jul 10;20(7):e0328214. doi: 10.1371/journal.pone.0328214 (PMC12244826; doi:10.1371/journal.pone.0328214)

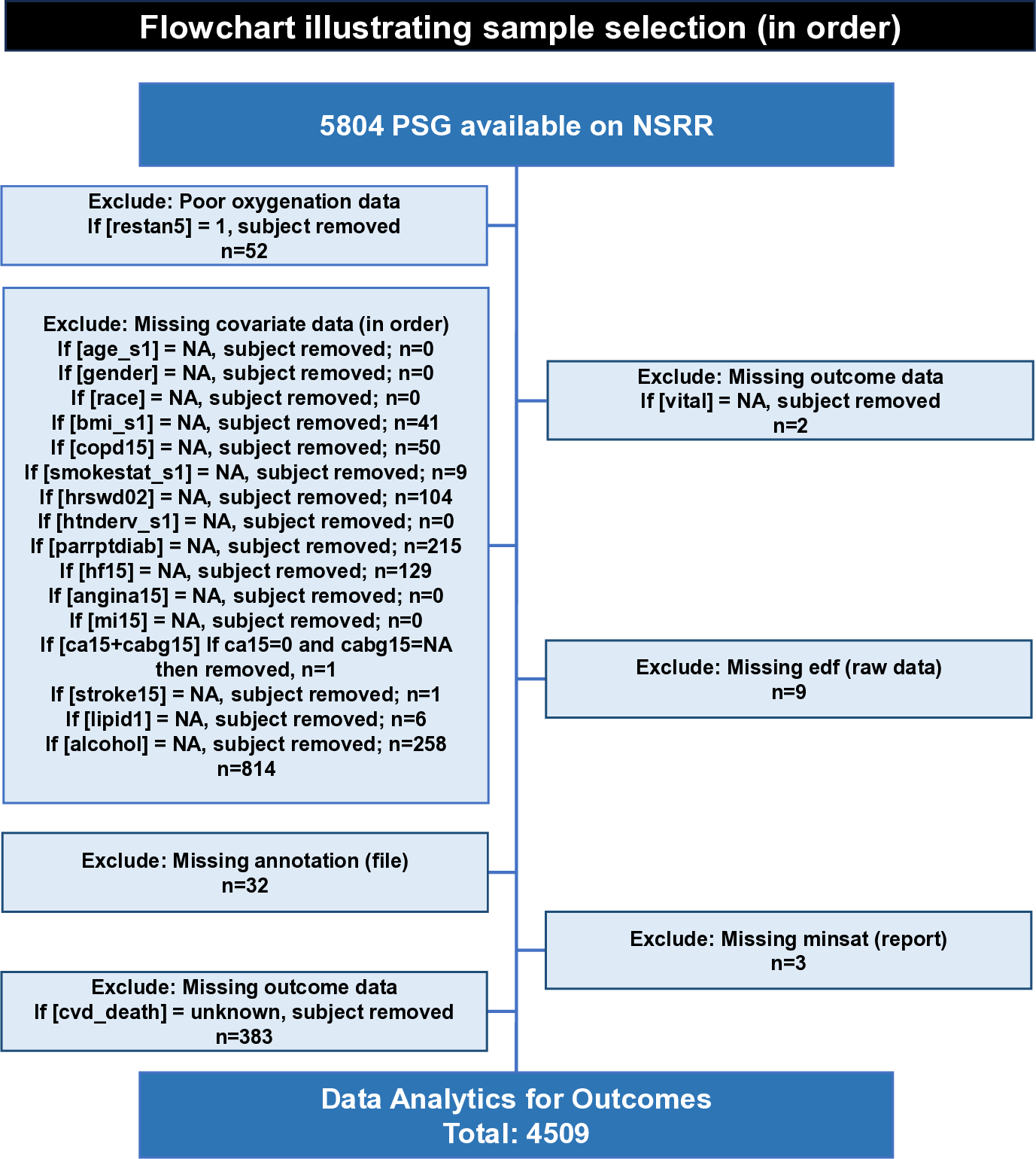

Supplement: S1 Fig — To ensure reproducibility, we list the variables utilized for subjects at baseline assessment (Visit 1) that were excluded. Poor oxygenation signals were excluded using the variable [restan5]; subjects missing all-cause mortality data were excluded using the variable [vital]; subjects missing covariate data were excluded in this order: age [age_s1], gender [gender], race [race], BMI [bmi_s1], COPD [copd15], smoking status [smokestat_s1], sleep duration [hrswd02], hypertension [htnderv_s1], diabetes [parrptdiab], congestive heart failure [hf15], angina [angina15], myocardial infarction [mi15], coronary revascularization [ca15 + cabg15], stroke [stroke15], lipid-lowering medication [lipid1], alcohol use [alcohol]; subjects with missing edf or annotation files or missing minsat on the report were excluded; subjects with missing cardiovascular mortality data were excluded using the variable [cvd_death]. (TIF) [file pone.0328214.s002.tif]

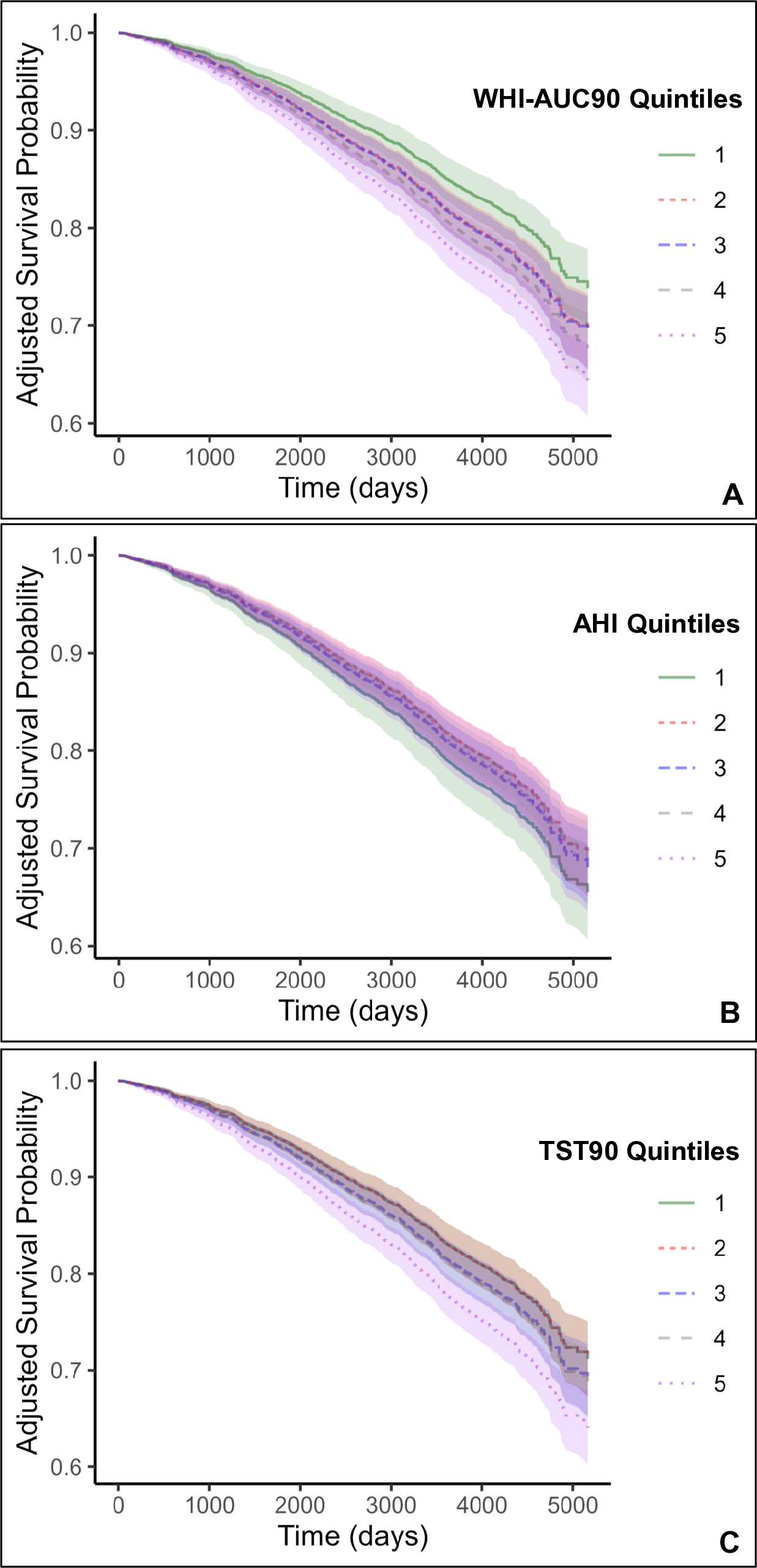

Supplement: S2 Fig — The survival probability for Model 4 (Hazard Ratios and confidence intervals in S1 Table) is presented for (A) WHI-AUC90, (B) AHI, and (C) TST90 models. Model 4 is adjusted for: (1) demographic covariates (age, gender, race, BMI, COPD, smoking, alcohol, sleep duration), (2) cardiometabolic covariates (diabetes, hypertension, congestive heart failure, angina, myocardial infarction, coronary revascularization, stroke, lipid-lowering medication), (3) AHI (for WHI-AUC90 and TST90 models), (4) TST90/minsat (for WHI-AUC90 and AHI models) and (5) WHI-AUC90 (for AHI and TST90 models). Adjusted survival curves were computed by averaging the predicted survival curves for each subject within the SHHS cohort. Note that among the survival curves by quintile the WHI-AUC90 demonstrates a coherent and logical progression specifically in comparison to AHI. (TIF) [file pone.0328214.s003.tif]
