# Supplementary material for: Weighted Hypoxemia Index: An adaptable method for quantifying hypoxemia severity
Source: PLoS One. 2025 Jul 10;20(7):e0328214. doi: 10.1371/journal.pone.0328214 (PMC12244826; doi:10.1371/journal.pone.0328214)
Supplement: S1 Table — (DOCX) [file pone.0328214.s004.docx]

|  | **WHI-AUC90** | **Hazard Ratio** | *P value* | **AHI** | **Hazard Ratio** | *P value* | **TST90** | **Hazard Ratio** | *P value* |
| --- | --- | --- | --- | --- | --- | --- | --- | --- | --- |
| **Model 0**  *Metric Alone* | **Q1** | 1 | *--* | **Q1** | 1 | *--* | **Q1** | 1 | *--* |
|  | **Q2** | 1.63 (1.29, 2.07) | ***<.001****** | **Q2** | 1.12 (0.91, 1.39) | *.292* | **Q2** | 0.94 (0.75, 1.18) | *.594* |
|  | **Q3** | 2.02 (1.61, 2.54) | ***<.001****** | **Q3** | 1.33 (1.08, 1.63) | ***.007***** | **Q3** | 1.37 (1.11, 1.68) | ***.003***** |
|  | **Q4** | 2.46 (1.97, 3.07) | ***<.001****** | **Q4** | 1.65 (1.35, 2.01) | ***<.001****** | **Q4** | 1.48 (1.21, 1.82) | ***<.001****** |
|  | **Q5** | 3.46 (2.79, 4.29) | ***<.001****** | **Q5** | 1.86 (1.53, 2.26) | ***<.001****** | **Q5** | 2.36 (1.95, 2.85) | ***<.001****** |
| **Model 1**  *Model 0 + Demographic^a^ +*  *Cardiometabolic^b^* | **Q1** | 1 | *--* | **Q1** | 1 | *--* | **Q1** | 1 | *--* |
|  | **Q2** | 1.27 (1.00, 1.62) | ***.047**** | **Q2** | 0.82 (0.66, 1.02) | *.078* | **Q2** | 1.00 (0.80, 1.25) | *.973* |
|  | **Q3** | 1.28 (1.02, 1.62) | ***.036**** | **Q3** | 0.89 (0.72, 1.10) | *.289* | **Q3** | 1.11 (0.90, 1.37) | *.332* |
|  | **Q4** | 1.37 (1.09, 1.72) | ***.007***** | **Q4** | 0.86 (0.70, 1.06) | *.166* | **Q4** | 1.11 (0.90, 1.37) | *.308* |
|  | **Q5** | 1.69 (1.35, 2.11) | ***<.001****** | **Q5** | 0.97 (0.78, 1.19) | *.756* | **Q5** | 1.46 (1.20, 1.79) | ***<.001****** |
| **Model 1A**  *Model 0 + Demographic^a^* | **Q1** | 1 | *--* | **Q1** | 1 | *--* | **Q1** | 1 | *--* |
|  | **Q2** | 1.32 (1.04, 1.68) | ***.022**** | **Q2** | 0.84 (0.67, 1.04) | *.103* | **Q2** | 1.02 (0.81, 1.27) | *.888* |
|  | **Q3** | 1.30 (1.03, 1.64) | ***.027**** | **Q3** | 0.92 (0.74, 1.13) | *.418* | **Q3** | 1.16 (0.94, 1.43) | *.172* |
|  | **Q4** | 1.41 (1.13, 1.77) | ***.003***** | **Q4** | 0.90 (0.73, 1.11) | *.333* | **Q4** | 1.14 (0.92, 1.40) | *.229* |
|  | **Q5** | 1.79 (1.43, 2.24) | ***<.001****** | **Q5** | 1.03 (0.84, 1.27) | *.780* | **Q5** | 1.54 (1.26, 1.88) | ***<.001****** |
| **Model 2A**  *Model 1A +*  *AHI^c^* | **Q1** | 1 | *--* | **Q1** | NA^f^ | *--* | **Q1** | 1 | *--* |
|  | **Q2** | 1.33 (1.05, 1.68) | ***.020**** | **Q2** | NA^f^ | *--* | **Q2** | 1.02 (0.81, 1.27) | *.871* |
|  | **Q3** | 1.31 (1.04, 1.65) | ***.024**** | **Q3** | NA^f^ | *--* | **Q3** | 1.17 (0.95, 1.44) | *.147* |
|  | **Q4** | 1.43 (1.14, 1.81) | ***.002***** | **Q4** | NA^f^ | *--* | **Q4** | 1.16 (0.94, 1.43) | *.179* |
|  | **Q5** | 1.84 (1.45, 2.34) | ***<.001****** | **Q5** | NA^f^ | *--* | **Q5** | 1.60 (1.28, 1.99) | ***<.001****** |
| **Model 2B**  *Model 1A +*  *{TST90+Min Sat}^d^* | **Q1** | 1 | *--* | **Q1** | 1 | *--* | **Q1** | NA^g^ | *--* |
|  | **Q2** | 1.34 (1.06, 1.71) | ***.015**** | **Q2** | 0.83 (0.67, 1.03) | *.084* | **Q2** | NA^g^ | *--* |
|  | **Q3** | 1.34 (1.06, 1.70) | ***.015**** | **Q3** | 0.89 (0.72, 1.10) | *.278* | **Q3** | NA^g^ | *--* |
|  | **Q4** | 1.48 (1.16, 1.89) | ***.001***** | **Q4** | 0.85 (0.69, 1.05) | *.136* | **Q4** | NA^g^ | *--* |
|  | **Q5** | 1.66 (1.26, 2.18) | ***<.001****** | **Q5** | 0.89 (0.70, 1.12) | *.317* | **Q5** | NA^g^ | *--* |
| **Model 3**  *Model 1A + AHI^c^+{TST90+*  *Min Sat}^d^+WHI*^e^ | **Q1** | 1 | *--* | **Q1** | 1 | *--* | **Q1** | 1 | *--* |
|  | **Q2** | 1.35 (1.06, 1.71) | ***.015**** | **Q2** | 0.83 (0.67, 1.04) | *.102* | **Q2** | 1.03 (0.82, 1.30) | *.774* |
|  | **Q3** | 1.34 (1.06, 1.70) | ***.015**** | **Q3** | 0.90 (0.72, 1.12) | *.335* | **Q3** | 1.20 (0.96, 1.50) | *.105* |
|  | **Q4** | 1.49 (1.17, 1.90) | ***.001***** | **Q4** | 0.87 (0.68, 1.11) | *.259* | **Q4** | 1.20 (0.95, 1.51) | *.135* |
|  | **Q5** | 1.67 (1.27, 2.21) | ***<.001****** | **Q5** | 0.93 (0.64, 1.36) | *.722* | **Q5** | 1.46 (1.11, 1.92) | ***.006***** |
| **Model 4**  *Model 3 + Cardiometabolic^b^* | **Q1** | 1 | *--* | **Q1** | 1 | *--* | **Q1** | 1 | *--* |
|  | **Q2** | 1.29 (1.02, 1.64) | ***.035**** | **Q2** | 0.82 (0.65, 1.02) | *.070* | **Q2** | 1.01 (0.80, 1.27) | *.937* |
|  | **Q3** | 1.32 (1.04, 1.67) | ***.022**** | **Q3** | 0.86 (0.69, 1.08) | *.194* | **Q3** | 1.14 (0.92, 1.43) | *.232* |
|  | **Q4** | 1.43 1.13, 1.83) | ***.004***** | **Q4** | 0.81 (0.63, 1.04) | *.094* | **Q4** | 1.17 (0.92, 1.48) | *.201* |
|  | **Q5** | 1.58 (1.20, 2.09) | ***.001***** | **Q5** | 0.84 (0.57, 1.23) | *.363* | **Q5** | 1.41 (1.07, 1.86) | ***.013**** |

**S1 Table. Weighted Hypoxemia Index predicts All-Cause Mortality better than TST90 and AHI.**

**WHI-AUC90,** Weighted Hypoxemia Index of Area Under the Curve set at upper threshold of 90%.

**AHI,** Apnea-hypopnea index, 3% criterion.

**TST90,** percent time of study with oxygen saturation below 90%.

**Min Sat,** minimum saturation.

**Model 0** is unadjusted hazard ratios (95% confidence intervals) for WHI-AUC90 vs AHI vs TST90.

**Models 1, 1A, 2A, 2B, 3 and 4** are adjusted hazard ratios (95% confidence intervals) for WHI-AUC90 vs AHI vs TST90.

**^a^Demographic covariates** include age, gender, race, BMI, COPD, smoking, alcohol, and sleep duration.

**^b^Cardiometabolic covariates** include diabetes, hypertension, congestive heart failure, angina, myocardial infarction, coronary revascularization, stroke, lipid-lowering medication.

^c^for WHI-AUC90 and TST90 (from SHHS report).

^d^for WHI-AUC90 and AHI (from SHHS report).

^e^for TST90 and AHI.

^f^ NA Since AHI is the metric, AHI was not adjusted.

^g^NA Since TST90 is the metric, TST90+Min Sat (from SHHS report) was not adjusted.

**P*<.05; ** *P*<.01; *** *P*<.001. Quintiles 2-5 are compared to Quintile 1.
