# Supplementary material for: Weighted Hypoxemia Index: An adaptable method for quantifying hypoxemia severity
Source: PLoS One. 2025 Jul 10;20(7):e0328214. doi: 10.1371/journal.pone.0328214 (PMC12244826; doi:10.1371/journal.pone.0328214)
Supplement: S2 Table — (DOCX) [file pone.0328214.s005.docx]

**S2 Table. Weighted Hypoxemia Index does not predict CVD mortality.**

|  | **WHI-AUC90** | **Hazard Ratio** | *p value* | **AHI** | **Hazard Ratio** | *p value* | **TST90** | **Hazard Ratio** | *p value* |
| --- | --- | --- | --- | --- | --- | --- | --- | --- | --- |
| **Model 0**  *Metric Alone* | **Q1** | 1 | *--* | **Q1** | 1 | *--* | **Q1** | 1 | *--* |
|  | **Q2** | 1.41 (0.93, 2.13) | *.109* | **Q2** | 1.61 (1.07, 2.43) | ***.023**** | **Q2** | 0.87 (0.58, 1.32) | *.510* |
|  | **Q3** | 1.94 (1.31, 2.88) | ***.001***** | **Q3** | 1.92 (1.29, 2.87) | ***.001***** | **Q3** | 1.61 (1.12, 2.31) | ***.011**** |
|  | **Q4** | 2.47 (1.68, 3.62) | ***<.001****** | **Q4** | 2.32 (1.57, 3.42) | ***<.001****** | **Q4** | 1.72 (1.20, 2.46) | ***.003***** |
|  | **Q5** | 3.12 (2.15, 4.54) | ***<.001****** | **Q5** | 2.60 (1.76, 3.82) | ***<.001****** | **Q5** | 2.37 (1.67, 3.37) | ***<.001****** |
| **Model 1**  *Model 0 + Demographic^a^ +*  *Cardiometabolic^b^* | **Q1** | 1 | *--* | **Q1** | 1 | *--* | **Q1** | 1 | *--* |
|  | **Q2** | 0.93 (0.61, 1.42) | *.733* | **Q2** | 0.86 (0.56, 1.31) | *.469* | **Q2** | 0.87 (0.57, 1.33) | *.533* |
|  | **Q3** | 1.03 (0.68, 1.55) | *.895* | **Q3** | 1.00 (0.67, 1.51) | *.998* | **Q3** | 1.16 (0.80, 1.69) | *.434* |
|  | **Q4** | 1.19 (0.81, 1.76) | *.383* | **Q4** | 0.86 (0.57, 1.31) | *.487* | **Q4** | 1.18 (0.81, 1.71) | *.384* |
|  | **Q5** | 1.26 (0.85, 1.88) | *.249* | **Q5** | 1.05 (0.69, 1.59) | *.816* | **Q5** | 1.29 (0.89, 1.88) | *.179* |
| **Model 1A**  *Model 0 + Demographic^a^* | **Q1** | 1 | *--* | **Q1** | 1 | *--* | **Q1** | 1 | *--* |
|  | **Q2** | 1.09 (0.72, 1.66) | *.685* | **Q2** | 0.92 (0.60, 1.39) | *.678* | **Q2** | 0.95 (0.63, 1.44) | *.802* |
|  | **Q3** | 1.01 (0.68, 1.51) | *.966* | **Q3** | 1.07 (0.71, 1.60) | *.760* | **Q3** | 1.23 (0.85, 1.78) | *.279* |
|  | **Q4** | 1.25 (0.85, 1.85) | *.256* | **Q4** | 0.98 (0.66, 1.47) | *.929* | **Q4** | 1.21 (0.84, 1.75) | *.312* |
|  | **Q5** | 1.46 (0.98, 2.16) | *.060* | **Q5** | 1.19 (0.79, 1.79) | *.399* | **Q5** | 1.48 (1.02, 2.14) | *.038** |
| **Model 2A**  *Model 1A +*  *AHI^c^* | **Q1** | 1 | *--* | **Q1** | NA^f^ | *--* | **Q1** | 1 | *--* |
|  | **Q2** | 1.09 (0.72, 1.66 | *.692* | **Q2** | NA^f^ | *--* | **Q2** | 0.95 (0.62, 1.44) | *.801* |
|  | **Q3** | 1.01 (0.67, 1.50) | *.976* | **Q3** | NA^f^ | *--* | **Q3** | 1.23 (0.84, 1.78) | *.286* |
|  | **Q4** | 1.24 (0.84, 1.85) | *.281* | **Q4** | NA^f^ | *--* | **Q4** | 1.21 (0.83, 1.76) | *.331* |
|  | **Q5** | 1.44 (0.95, 2.19) | *.089* | **Q5** | NA^f^ | *--* | **Q5** | 1.47 (0.98, 2.20) | *.064* |
| **Model 2B**  *Model 1A +*  *{TST90+Min Sat}^d^* | **Q1** | 1 | *--* | **Q1** | 1 | *--* | **Q1** | NA^g^ | *--* |
|  | **Q2** | 1.09 (0.72, 1.66) | *.687* | **Q2** | 0.89 (0.59, 1.36) | *.601* | **Q2** | NA^g^ | *--* |
|  | **Q3** | 1.01 (0.67, 1.52) | *.971* | **Q3** | 1.02 (0.67, 1.54) | *.938* | **Q3** | NA^g^ | *--* |
|  | **Q4** | 1.24 (0.81, 1.88) | *.324* | **Q4** | 0.92 (0.61, 1.40) | *.704* | **Q4** | NA^g^ | *--* |
|  | **Q5** | 1.17 (0.71, 1.92) | *.529* | **Q5** | 1.01 (0.64, 1.58) | *.971* | **Q5** | NA^g^ | *--* |
| **Model 3**  *Model 1A + AHI^c^+{TST90+*  *Min Sat}^d^+WHI*^e^ | **Q1** | 1 | *--* | **Q1** | 1 | *--* | **Q1** | 1 | *--* |
|  | **Q2** | 1.09 (0.72, 1.66) | *.685* | **Q2** | 0.90 (0.59, 1.37) | *.623* | **Q2** | 0.95 (0.62, 1.44) | *.800* |
|  | **Q3** | 1.01 (0.67, 1.52) | *.970* | **Q3** | 1.02 (0.66, 1.57) | *.937* | **Q3** | 1.23 (0.83, 1.83) | *.298* |
|  | **Q4** | 1.24 (0.81, 1.89) | *.320* | **Q4** | 0.95 (0.59, 1.54) | *.848* | **Q4** | 1.20 (0.79, 1.83) | *.389* |
|  | **Q5** | 1.18 (0.71, 1.94) | *.522* | **Q5** | 1.10 (0.54, 2.26) | *.787* | **Q5** | 1.24 (0.74, 2.07) | *.411* |
| **Model 4**  *Model 3 + Cardiometabolic^b^* | **Q1** | 1 | *--* | **Q1** | 1 | *--* | **Q1** | 1 | *--* |
|  | **Q2** | 0.92 (0.60, 1.41) | *.708* | **Q2** | 0.84 (0.55, 1.30) | *.441* | **Q2** | 0.86 (0.56, 1.32) | *.488* |
|  | **Q3** | 1.01 (0.67, 1.54) | *.955* | **Q3** | 0.98 (0.63, 1.51) | *.924* | **Q3** | 1.15 (0.77, 1.70) | *.493* |
|  | **Q4** | 1.15 (0.76, 1.76) | *.508* | **Q4** | 0.87 (0.53, 1.41) | *.564* | **Q4** | 1.15 (0.76, 1.74) | *.508* |
|  | **Q5** | 1.00 (0.61, 1.66) | *.988* | **Q5** | 1.05 (0.51, 2.17) | *.891* | **Q5** | 1.08 (0.65, 1.80) | *.774* |

WHI-AUC90, Weighted Hypoxemia Index of Area Under the Curve set at upper threshold of 90%; AHI, Apnea-hypopnea index, 3% criterion; TST90, percent time of study with oxygen saturation below 90%; Min Sat, minimum saturation.

Model 0 is unadjusted hazard ratios (95% confidence intervals) for WHI-AUC90 vs AHI vs TST90.

Models 1, 1A, 2A, 2B, 3 and 4 are adjusted hazard ratios (95% confidence intervals) for WHI-AUC90 vs AHI vs TST90.

^a^Demographic covariates include age, gender, race, BMI, COPD, smoking, alcohol, sleep duration.

^b^Cardiometabolic covariates include diabetes, hypertension, congestive heart failure, angina, myocardial infarction, coronary revascularization, stroke, lipid-lowering medication.

^c^ for WHI-AUC90 and TST90 (from SHHS report).

^d^ for WHI-AUC90 and AHI (from SHHS report).

^e^ for TST90 and AHI.

^f^ NA Since AHI is the metric, AHI was not adjusted.

^g^ NA Since TST90 is the metric, TST90+Min Sat (from SHHS report) was not adjusted.

*p<.05; ** p<.01; *** p<.001. Quintiles 2-5 are compared to Quintile 1.
