# Supplementary material for: Weighted Hypoxemia Index: An adaptable method for quantifying hypoxemia severity
Source: PLoS One. 2025 Jul 10;20(7):e0328214. doi: 10.1371/journal.pone.0328214 (PMC12244826; doi:10.1371/journal.pone.0328214)
Supplement: S3 Table — (DOCX) [file pone.0328214.s006.docx]

**S3 Table. Model 1 predicting CVD mortality, comparison of weighted vs unweighted and AUC vs AAC at different upper thresholds.**

| **Weighted** | **Quintiles** | **Hazard Ratio** | ***P value*** | **Unweighted** | **Quintiles** | **Hazard Ratio** | ***P value*** |
| --- | --- | --- | --- | --- | --- | --- | --- |
| **WHI**  **AUC**  **92** | **Q1** | 1 | *--* | **AUC**  **92** | **Q1** | 1 | *--* |
|  | **Q2** | 1.23 (0.75, 2.01) | *.407* |  | **Q2** | 1.32 (0.82, 2.13) | *.256* |
|  | **Q3** | 1.36 (0.85, 2.19) | *.204* |  | **Q3** | 1.26 (0.79, 2.01) | *.339* |
|  | **Q4** | 1.04 (0.63, 1.71) | *.874* |  | **Q4** | 1.07 (0.66, 1.74) | *.780* |
|  | **Q5** | 1.44 (0.89, 2.32) | *.140* |  | **Q5** | 1.27 (0.79, 2.05) | *.318* |
| **WHI**  **AAC**  **92** | **Q1** | 1 | *--* | **AAC**  **92** | **Q1** | 1 | *--* |
|  | **Q2** | 0.96 (0.60, 1.54) | *.873* |  | **Q2** | 1.03 (0.64, 1.64) | *.911* |
|  | **Q3** | 1.25 (0.80, 1.95) | *.334* |  | **Q3** | 1.31 (0.84, 2.06) | *.235* |
|  | **Q4** | 0.95 (0.59, 1.51) | *.819* |  | **Q4** | 1.08 (0.68, 1.71) | *.752* |
|  | **Q5** | 1.20 (0.77, 1.89) | *.421* |  | **Q5** | 1.24 (0.79, 1.94) | *.360* |
| **WHI**  **AUC**  **90** | **Q1** | 1 | *--* | **AUC**  **90** | **Q1** | 1 | *--* |
|  | **Q2** | 0.93 (0.61, 1.42) | *.733* |  | **Q2** | 0.89 (0.58, 1.35) | *.580* |
|  | **Q3** | 1.03 (0.68, 1.55) | *.895* |  | **Q3** | 1.10 (0.74, 1.64) | *.635* |
|  | **Q4** | 1.19 (0.81, 1.76) | *.383* |  | **Q4** | 1.11 (0.75, 1.64) | *.586* |
|  | **Q5** | 1.26 (0.85, 1.88) | *.249* |  | **Q5** | 1.22 (0.82, 1.81) | *.325* |
| **WHI**  **AAC**  **90** | **Q1** | 1 | *--* | **AAC**  **90** | **Q1** | 1 | *--* |
|  | **Q2** | 0.95 (0.62, 1.46) | *.833* |  | **Q2** | 0.85 (0.56, 1.30) | *.451* |
|  | **Q3** | 1.18 (0.79, 1.76) | *.422* |  | **Q3** | 1.09 (0.74, 1.62) | *.657* |
|  | **Q4** | 1.19 (0.80, 1.76) | *.384* |  | **Q4** | 1.13 (0.77, 1.66) | *.539* |
|  | **Q5** | 1.36 (0.91, 2.02) | *.133* |  | **Q5** | 1.15 (0.77, 1.71) | *.485* |
| **WHI**  **AUC**  **88** | **Q1** | 1 | *--* | **AUC**  **88** | **Q1** | 1 | *--* |
|  | **Q2** | 1.28 (0.83, 1.98) | *.261* |  | **Q2** | 1.24 (0.80, 1.91) | *.344* |
|  | **Q3** | 1.27 (0.85, 1.90) | *.243* |  | **Q3** | 1.43 (0.96, 2.13) | *.078* |
|  | **Q4** | 1.53 (1.05, 2.24) | ***.028**** |  | **Q4** | 1.35 (0.92, 1.99) | *.125* |
|  | **Q5** | 1.48 (1.01, 2.18) | ***.045**** |  | **Q5** | 1.57 (1.07, 2.30) | ***.021**** |
| **WHI**  **AAC**  **88** | **Q1** | 1 | *--* | **AAC**  **88** | **Q1** | 1 | *--* |
|  | **Q2** | 1.29 (0.84, 1.97) | *.238* |  | **Q2** | 1.29 (0.84, 1.98) | *.245* |
|  | **Q3** | 1.30 (0.87, 1.94) | *.202* |  | **Q3** | 1.38 (0.93, 2.04) | *.112* |
|  | **Q4** | 1.51 (1.03, 2.22) | ***.036**** |  | **Q4** | 1.43 (0.97, 2.10) | *.073* |
|  | **Q5** | 1.49 (1.02, 2.19) | ***.040**** |  | **Q5** | 1.50 (1.02, 2.20) | ***.040**** |
| **WHI**  **AUC**  **86** | **Q1** | 1 | *--* | **AUC**  **86** | **Q1** | 1 | *--* |
|  | **Q2** | 0.76 (0.42, 1.37) | *.364* |  | **Q2** | 0.76 (0.42, 1.37) | *.363* |
|  | **Q3** | 1.20 (0.86, 1.66) | *.289* |  | **Q3** | 1.22 (0.88, 1.69) | *.240* |
|  | **Q4** | 1.21 (0.87, 1.67) | *.259* |  | **Q4** | 1.23 (0.88, 1.70) | *.222* |
|  | **Q5** | 1.38 (1.01, 1.90) | ***.045**** |  | **Q5** | 1.33 (0.97, 1.83) | *.074* |
| **WHI**  **AAC**  **86** | **Q1** | 1 | *--* | **AAC**  **86** | **Q1** | 1 | *--* |
|  | **Q2** | 0.76 (0.42, 1.37) | *.362* |  | **Q2** | 0.76 (0.42, 1.37) | *.359* |
|  | **Q3** | 1.16 (0.83, 1.61) | *.388* |  | **Q3** | 1.18 (0.85, 1.63) | *.332* |
|  | **Q4** | 1.21 (0.87, 1.68) | *.267* |  | **Q4** | 1.20 (0.86, 1.67) | *.285* |
|  | **Q5** | 1.42 (1.04, 1.95) | ***.029**** |  | **Q5** | 1.41 (1.03, 1.93) | ***.034**** |

**WHI** are metrics that are weighted using a linear weighted factor of duration of desaturation/resaturation event.

**AUC** are metrics where area is calculated under the S_p_O_2_ curve.

**AAC** are metrics where area is calculated above the S_p_O_2_ curve.

**The number** following AUC/AAC is the upper threshold, e.g., AAC88 is the area above the curve below an upper threshold of 88%.

**Model 1** uses the same 4509 subjects characterized in Table 1: Hazard ratios (95% confidence intervals) controlled for demographic covariates (age, gender, race, BMI, COPD, smoking, alcohol, sleep duration) and cardiometabolic covariates (diabetes, hypertension, congestive heart failure, angina, myocardial infarction, coronary revascularization, stroke, lipid-lowering medication).

**P*<.05; ** *P*<.01; *** *P*<.001. Quintiles 2-5 are compared to Quintile 1.
