# Supplementary material for: Weighted Hypoxemia Index: An adaptable method for quantifying hypoxemia severity
Source: PLoS One. 2025 Jul 10;20(7):e0328214. doi: 10.1371/journal.pone.0328214 (PMC12244826; doi:10.1371/journal.pone.0328214)
Supplement: S4 Table — (DOCX) [file pone.0328214.s007.docx]

**S4 Table. Benjamini-Hochberg correction of Table 3.**

| **Weighted** | **Quintiles** | ***P value*** | **Unweighted** | **Quintiles** | ***P value*** |
| --- | --- | --- | --- | --- | --- |
| **WHI**  **AUC**  **92** | **Q1** | NA | **AUC**  **92** | **Q1** | NA |
|  | **Q2** | .120 |  | **Q2** | .031* |
|  | **Q3** | .165 |  | **Q3** | .090 |
|  | **Q4** | .333 |  | **Q4** | .102 |
|  | **Q5** | <.001*** |  | **Q5** | <.001*** |
| **WHI**  **AAC**  **92** | **Q1** | NA | **AAC**  **92** | **Q1** | NA |
|  | **Q2** | .030* |  | **Q2** | .042* |
|  | **Q3** | .036* |  | **Q3** | .054 |
|  | **Q4** | .094 |  | **Q4** | .054 |
|  | **Q5** | <.001*** |  | **Q5** | <.001*** |
| **WHI**  **AUC**  **90** | **Q1** | NA | **AUC**  **90** | **Q1** | NA |
|  | **Q2** | .060 |  | **Q2** | .074 |
|  | **Q3** | .051 |  | **Q3** | .048* |
|  | **Q4** | .014* |  | **Q4** | .021* |
|  | **Q5** | <.001*** |  | **Q5** | <.001*** |
| **WHI**  **AAC**  **90** | **Q1** | NA | **AAC**  **90** | **Q1** | NA |
|  | **Q2** | .067 |  | **Q2** | .078 |
|  | **Q3** | .013* |  | **Q3** | .031* |
|  | **Q4** | .012* |  | **Q4** | .014* |
|  | **Q5** | <.001*** |  | **Q5** | <.001*** |
| **WHI**  **AUC**  **88** | **Q1** | NA | **AUC**  **88** | **Q1** | NA |
|  | **Q2** | .042* |  | **Q2** | .018* |
|  | **Q3** | .002** |  | **Q3** | .005** |
|  | **Q4** | <.001*** |  | **Q4** | <.001*** |
|  | **Q5** | <.001*** |  | **Q5** | <.001*** |
| **WHI**  **AAC**  **88** | **Q1** | NA | **AAC**  **88** | **Q1** | NA |
|  | **Q2** | .038* |  | **Q2** | .030* |
|  | **Q3** | .002** |  | **Q3** | .002** |
|  | **Q4** | <.001*** |  | **Q4** | <.001*** |
|  | **Q5** | <.001*** |  | **Q5** | <.001*** |
| **WHI**  **AUC**  **86** | **Q1** | NA | **AUC**  **86** | **Q1** | NA |
|  | **Q2** | .186 |  | **Q2** | .186 |
|  | **Q3** | .060 |  | **Q3** | .054 |
|  | **Q4** | .012* |  | **Q4** | .014* |
|  | **Q5** | <.001*** |  | **Q5** | <.001*** |
| **WHI**  **AAC**  **86** | **Q1** | NA | **AAC**  **86** | **Q1** | NA |
|  | **Q2** | .186 |  | **Q2** | .186 |
|  | **Q3** | .095 |  | **Q3** | .042* |
|  | **Q4** | .007** |  | **Q4** | .014* |
|  | **Q5** | <.001*** |  | **Q5** | <.001*** |

* P-values were adjusted for multiple comparisons using the Benjamini-Hochberg procedure

(FDR = 0.05) across 64 tests (Q2–Q5 for 8 combinations of signal × metric × weighting).
