# Supplementary material for: Weighted Hypoxemia Index: An adaptable method for quantifying hypoxemia severity
Source: PLoS One. 2025 Jul 10;20(7):e0328214. doi: 10.1371/journal.pone.0328214 (PMC12244826; doi:10.1371/journal.pone.0328214)
Supplement: S5 Table — (DOCX) [file pone.0328214.s008.docx]

**S5 Table. Benjamini-Hochberg correction of S1 Table.**

|  | **WHI-AUC90** | *P value* | **AHI** | *P value* | **TST90** | *P value* |
| --- | --- | --- | --- | --- | --- | --- |
| **Model 0**  *Metric Alone* | **Q1** | NA | **Q1** | NA | **Q1** | NA |
|  | **Q2** | .091 | **Q2** | .369 | **Q2** | .663 |
|  | **Q3** | .074 | **Q3** | .023* | **Q3** | .012* |
|  | **Q4** | .021* | **Q4** | <.001*** | **Q4** | .001** |
|  | **Q5** | <.001*** | **Q5** | <.001*** | **Q5** | <.001*** |
| **Model 1**  *Model 0 + Demographic^a^ +*  *Cardiometabolic^b^* | **Q1** | NA | **Q1** | NA | **Q1** | NA |
|  | **Q2** | .052 | **Q2** | .144 | **Q2** | .973 |
|  | **Q3** | .060 | **Q3** | .369 | **Q3** | .391 |
|  | **Q4** | .012* | **Q4** | .252 | **Q4** | .384 |
|  | **Q5** | <.001*** | **Q5** | .821 | **Q5** | .001** |
| **Model 1A**  *Model 0 + Demographic^a^* | **Q1** | NA | **Q1** | NA | **Q1** | NA |
|  | **Q2** | .051 | **Q2** | .174 | **Q2** | .912 |
|  | **Q3** | .055 | **Q3** | .474 | **Q3** | .257 |
|  | **Q4** | .010** | **Q4** | .391 | **Q4** | .315 |
|  | **Q5** | <.001*** | **Q5** | .823 | **Q5** | <.001*** |
| **Model 2A**  *Model 1A +*  *AHI^c^* | **Q1** | NA | **Q1** | NA | **Q1** | NA |
|  | **Q2** | .040* | **Q2** | NA | **Q2** | .907 |
|  | **Q3** | .040* | **Q3** | NA | **Q3** | .228 |
|  | **Q4** | .007** | **Q4** | NA | **Q4** | .262 |
|  | **Q5** | .002** | **Q5** | NA | **Q5** | <.001*** |
| **Model 2B**  *Model 1A +*  *{TST90+Min Sat}^d^* | **Q1** | NA | **Q1** | NA | **Q1** | NA |
|  | **Q2** | .091 | **Q2** | .152 | **Q2** | NA |
|  | **Q3** | .074 | **Q3** | .364 | **Q3** | NA |
|  | **Q4** | .021* | **Q4** | .215 | **Q4** | NA |
|  | **Q5** | <.001*** | **Q5** | .388 | **Q5** | NA |
| **Model 3**  *Model 1A + AHI^c^+{TST90+*  *Min Sat}^d^+WHI*^e^ | **Q1** | NA | **Q1** | NA | **Q1** | NA |
|  | **Q2** | .040* | **Q2** | .174 | **Q2** | .823 |
|  | **Q3** | .040* | **Q3** | .391 | **Q3** | .174 |
|  | **Q4** | .006** | **Q4** | .345 | **Q4** | .215 |
|  | **Q5** | .002** | **Q5** | .795 | **Q5** | .021* |
| **Model 4**  *Model 3 + Cardiometabolic^b^* | **Q1** | NA | **Q1** | NA | **Q1** | NA |
|  | **Q2** | .074 | **Q2** | .133 | **Q2** | .949 |
|  | **Q3** | .052 | **Q3** | .278 | **Q3** | .315 |
|  | **Q4** | .014* | **Q4** | .166 | **Q4** | .282 |
|  | **Q5** | .006** | **Q5** | .418 | **Q5** | .040* |

* P-values were adjusted for multiple comparisons using the Benjamini-Hochberg procedure

(FDR = 0.05) across 76 tests
