# Supplementary material for: Weighted Hypoxemia Index: An adaptable method for quantifying hypoxemia severity
Source: PLoS One. 2025 Jul 10;20(7):e0328214. doi: 10.1371/journal.pone.0328214 (PMC12244826; doi:10.1371/journal.pone.0328214)
Supplement: S6 Table — (DOCX) [file pone.0328214.s009.docx]

**S6 Table. Benjamini-Hochberg correction of S2 Table.**

|  | **WHI-AUC90** | *p value* | **AHI** | *p value* | **TST90** | *p value* |
| --- | --- | --- | --- | --- | --- | --- |
| **Model 0**  *Metric Alone* | **Q1** | NA | **Q1** | NA | **Q1** | NA |
|  | **Q2** | .552 | **Q2** | .173 | **Q2** | .941 |
|  | **Q3** | .013* | **Q3** | .015* | **Q3** | .091 |
|  | **Q4** | <.001*** | **Q4** | <.001*** | **Q4** | .033* |
|  | **Q5** | <.001*** | **Q5** | <.001*** | **Q5** | <.001*** |
| **Model 1**  *Model 0 + Demographic^a^ +*  *Cardiometabolic^b^* | **Q1** | NA | **Q1** | NA | **Q1** | NA |
|  | **Q2** | .998 | **Q2** | .941 | **Q2** | .941 |
|  | **Q3** | .998 | **Q3** | .998 | **Q3** | .941 |
|  | **Q4** | .941 | **Q4** | .941 | **Q4** | .941 |
|  | **Q5** | .941 | **Q5** | .998 | **Q5** | .849 |
| **Model 1A**  *Model 0 + Demographic^a^* | **Q1** | NA | **Q1** | NA | **Q1** | NA |
|  | **Q2** | .998 | **Q2** | .998 | **Q2** | .998 |
|  | **Q3** | .998 | **Q3** | .998 | **Q3** | .941 |
|  | **Q4** | .941 | **Q4** | .998 | **Q4** | .941 |
|  | **Q5** | .375 | **Q5** | .941 | **Q5** | .263 |
| **Model 2A**  *Model 1A +*  *AHI^c^* | **Q1** | NA | **Q1** | NA | **Q1** | NA |
|  | **Q2** | .998 | **Q2** | NA | **Q2** | .998 |
|  | **Q3** | .998 | **Q3** | NA | **Q3** | .941 |
|  | **Q4** | .941 | **Q4** | NA | **Q4** | .941 |
|  | **Q5** | .484 | **Q5** | NA | **Q5** | .375 |
| **Model 2B**  *Model 1A +*  *{TST90+Min Sat}^d^* | **Q1** | NA | **Q1** | NA | **Q1** | NA |
|  | **Q2** | .998 | **Q2** | .998 | **Q2** | NA |
|  | **Q3** | .998 | **Q3** | .998 | **Q3** | NA |
|  | **Q4** | .941 | **Q4** | .998 | **Q4** | NA |
|  | **Q5** | .941 | **Q5** | .998 | **Q5** | NA |
| **Model 3**  *Model 1A + AHI^c^+{TST90+*  *Min Sat}^d^+WHI*^e^ | **Q1** | NA | **Q1** | NA | **Q1** | NA |
|  | **Q2** | .998 | **Q2** | .998 | **Q2** | .998 |
|  | **Q3** | .998 | **Q3** | .998 | **Q3** | .941 |
|  | **Q4** | .941 | **Q4** | .998 | **Q4** | .941 |
|  | **Q5** | .941 | **Q5** | .998 | **Q5** | .941 |
| **Model 4**  *Model 3 + Cardiometabolic^b^* | **Q1** | NA | **Q1** | NA | **Q1** | NA |
|  | **Q2** | .998 | **Q2** | .941 | **Q2** | .941 |
|  | **Q3** | .998 | **Q3** | .998 | **Q3** | .941 |
|  | **Q4** | .941 | **Q4** | .974 | **Q4** | .941 |
|  | **Q5** | .998 | **Q5** | .998 | **Q5** | .998 |

* P-values were adjusted for multiple comparisons using the Benjamini-Hochberg procedure

(FDR = 0.05) across 76 tests
