# Supplementary material for: Weighted Hypoxemia Index: An adaptable method for quantifying hypoxemia severity
Source: PLoS One. 2025 Jul 10;20(7):e0328214. doi: 10.1371/journal.pone.0328214 (PMC12244826; doi:10.1371/journal.pone.0328214)
Supplement: S7 Table — (DOCX) [file pone.0328214.s010.docx]

**S7 Table. Benjamini-Hochberg correction of S3 Table.**

| **Weighted** | **Quintiles** | ***P value*** | **Unweighted** | **Quintiles** | ***P value*** |
| --- | --- | --- | --- | --- | --- |
| **WHI**  **AUC**  **92** | **Q1** | NA | **AUC**  **92** | **Q1** | NA |
|  | **Q2** | .562 |  | **Q2** | .552 |
|  | **Q3** | .552 |  | **Q3** | .552 |
|  | **Q4** | .903 |  | **Q4** | .861 |
|  | **Q5** | .552 |  | **Q5** | .552 |
| **WHI**  **AAC**  **92** | **Q1** | NA | **AAC**  **92** | **Q1** | NA |
|  | **Q2** | .903 |  | **Q2** | .911 |
|  | **Q3** | .552 |  | **Q3** | .552 |
|  | **Q4** | .888 |  | **Q4** | .845 |
|  | **Q5** | .562 |  | **Q5** | .552 |
| **WHI**  **AUC**  **90** | **Q1** | NA | **AUC**  **90** | **Q1** | NA |
|  | **Q2** | .837 |  | **Q2** | .708 |
|  | **Q3** | .909 |  | **Q3** | .752 |
|  | **Q4** | .552 |  | **Q4** | .708 |
|  | **Q5** | .552 |  | **Q5** | .552 |
| **WHI**  **AAC**  **90** | **Q1** | NA | **AAC**  **90** | **Q1** | NA |
|  | **Q2** | .888 |  | **Q2** | .589 |
|  | **Q3** | .562 |  | **Q3** | .765 |
|  | **Q4** | .552 |  | **Q4** | .676 |
|  | **Q5** | .552 |  | **Q5** | .621 |
| **WHI**  **AUC**  **88** | **Q1** | NA | **AUC**  **88** | **Q1** | NA |
|  | **Q2** | .552 |  | **Q2** | .552 |
|  | **Q3** | .552 |  | **Q3** | .417 |
|  | **Q4** | .323 |  | **Q4** | .552 |
|  | **Q5** | .323 |  | **Q5** | .323 |
| **WHI**  **AAC**  **88** | **Q1** | NA | **AAC**  **88** | **Q1** | NA |
|  | **Q2** | .552 |  | **Q2** | .552 |
|  | **Q3** | .552 |  | **Q3** | .551 |
|  | **Q4** | .323 |  | **Q4** | .417 |
|  | **Q5** | .323 |  | **Q5** | .323 |
| **WHI**  **AUC**  **86** | **Q1** | NA | **AUC**  **86** | **Q1** | NA |
|  | **Q2** | .552 |  | **Q2** | .552 |
|  | **Q3** | .552 |  | **Q3** | .552 |
|  | **Q4** | .552 |  | **Q4** | .552 |
|  | **Q5** | .323 |  | **Q5** | .417 |
| **WHI**  **AAC**  **86** | **Q1** | NA | **AAC**  **86** | **Q1** | NA |
|  | **Q2** | .552 |  | **Q2** | .552 |
|  | **Q3** | .552 |  | **Q3** | .552 |
|  | **Q4** | .552 |  | **Q4** | .552 |
|  | **Q5** | .323 |  | **Q5** | .323 |

* P-values were adjusted for multiple comparisons using the Benjamini-Hochberg procedure

(FDR = 0.05) across 64 tests
